# Reduced miR-16 levels are associated with VEGF upregulation in high-risk myelodysplastic syndromes

**DOI:** 10.7150/jca.52455

**Published:** 2021-01-30

**Authors:** Bei Xiong, Yanbo Nie, Yalan Yu, Shixuan Wang, Xuelan Zuo

**Affiliations:** 1 Department of Hematology, Zhongnan Hospital of Wuhan University, Wuhan, China.; 2 Sino-us-diagnostics, Tianjin, China.; 3 Department of Hematology, The First Affiliated Hospital of Nanchang University, Nanchang, China.

**Keywords:** myelodysplastic syndromes, vascular endothelial growth factor, miR-16, angiogenesis, SKM-1 cells.

## Abstract

**Objective:** Overexpression of vascular endothelial growth factor (VEGF), a major angiogenic factor, was found in myelodysplastic syndromes (MDS) and showed different expression statuses in different risk groups of MDS. We aimed to investigate the possible role of microRNA (miR)-15a and miR-16 on the regulation of VEGF expression and their effect on angiogenesis in lower- and higher-risk MDS.

**Methods:** We studied peripheral blood and bone marrow samples of MDS patients or several leukaemia and MDS cell lines by enzyme-linked immunosorbent assay, immunohistochemical staining, immunofluorescence and quantitative PCR for expression levels of VEGF, miR-15a and miR-16. MiRNA transfection and Luciferase reporter assays were conducted to investigate whether VEGF is a target of miR-16. Migration and tube formation assays were performed in cells exposed to medium from cells with overexpressed or knockdown miR-16.

**Results:** It showed a significantly lower level of miR-16 in higher-risk MDS patients, while the VEGF levels were upregulated. Inverse correlation between VEGF and miR-16 were determined in cells lines including SKM-1, THP-1, and K562 cells. Overexpression of miR-16 in SKM-1 cells resulted in reduced VEGF secretion and cell protein levels. Direct binding of miR-16 to the 3' untranslated region (3'-UTR) of VEGF was confirmed by luciferase reporter assays. The migration and tube formation of human umbilical vein endothelial cells decreased in the presence of medium from SKM-1 cells with overexpressed miR-16.

**Conclusion:** These data suggest that miR-16 may play a role in angiogenesis in higher-risk MDS by targeting VEGF and therefore modulating MDS progression. MiR-16 might be a novel therapeutic target in higher-risk MDS.

## Introduction

Myelodysplastic syndromes (MDS) are a heterogeneous group of haematological disorders characterised by dysplastic and ineffective hematopoiesis, peripheral cytopenias, and increased risk of transformation to acute myeloid leukaemia (AML) 1, 2. The prognosis varies a lot, ranging from a few months to more than 10 years 3, 4. Low-risk MDS often show hypercellularity in bone marrow and display increased apoptosis of haematopoietic progenitors. In contrast, high-risk MDS always link to a reduced level of apoptosis 5-7. Vascular endothelial growth factor (VEGF), a chief angiogenic factor, has attracted much attention for its critical role involved in regulating haematopoietic cells in addition to its role in angiogenesis and vasculogenesis 8, 9. Its deregulation promotes tumour proliferation and angiogenesis by stimulating endothelial as well as leukemic cells 10.

Several studies using different methods have revealed that VEGF is aberrantly expressed in low- and high-risk MDS 11-14. By western blotting and radioimmunoassay, Verstovstek *et al.* showed that increased VEGF expression in bone marrow samples of MDS patients is associated with decreased survival 11. Wimazal *et al.* demonstrated the correlation among the expression of VEGF mRNA, the percentage of immature myeloid cells, and the French- American-British (FAB) classification in the bone marrow of MDS patients 12. Gianelli *et al.* revealed a higher expression of VEGF levels normalised for bone marrow cellularity (VEGF index [VEGFi]) in the “very high risk” group than in the “very low risk” group according to the World Health Organization (WHO) classification-based prognostic scoring system (WPSS) by immunohistochemistry. High VEGFi predicted transfusion dependence and was inversely correlated with overall survival and leukaemia-free survival 13. In addition, Bellamy *et al.* demonstrated a higher immunohistochemical expression of VEGF receptors in the bone marrow of high-risk MDS than in low-risk MDS patients and healthy controls 14. The strong immunohistochemical co-expression of VEGF and its receptors in myeloblasts and immature myeloid elements of MDS bone marrow indicated that an autocrine loop of VEGF may facilitate self-renewal of leukaemia progenitors 14. These findings illustrate that, whereas apoptosis is increased in MDS patients with low-risk, the disease with progression into high-risk stage exhibit an acquired resistance to apoptosis and aberrant expression of VEGF. Therefore, we decided to investigate the mechanism of the dysregulation of VEGF in MDS and whether it contributes to disease progression. The recognition of the differences in molecular pathways between low- and high-risk MDS will hopefully provide important information in future targeted therapies.

MicroRNAs (miRNAs) are endogenous, single stranded molecules of about 22 nucleotides in length, which attract widespread attention for their significant regulatory roles in various biological processes 15-17. Emerging evidences have demonstrated aberrant expression of miRNAs in hematologic cancers, suggesting possible roles for miRNAs involved in haematopoiesis and tumorigenesis 18-20. We hypothesised that changes in the levels of one or more miRNAs might be involved in the increased expression of VEGF in high-risk MDS. Few studies have investigated the role of miRNAs in VEGF expression. A small number of studies have shown that microRNA (miR)-15a and miR-16 inhibit VEGF expression in lymphoma and multiple myeloma cell lines 21, 22, but whether these miRNAs contribute to the regulation of VEGF in MDS has not been studied yet. In this report, we investigated the expression status of miR-15a and miR-16 in MDS and their correlation with disease stages, and verified the possible role of miRNAs on the regulation of VEGF expression and their effect on angiogenesis in MDS.

## Materials and methods

### Cells and cell culture

Bone marrow samples from 80 new cases of MDS and 20 healthy controls at the Hematology Department of Zhongnan Hospital of Wuhan University were collected, followed by isolation of bone marrow mononuclear cells using Ficoll-Hypaque (Haoyang Biotechnology, Tianjin, China) gradient centrifugation. Then the enrichment of CD34+ cells from bone marrow mononuclear cells were performed according to a standard protocol using a CD34+ MicroBead kit (Miltenyi Biotec, Auburn, CA, USA). The purity of CD34+ cells was ≥90% by fluorescence-activated cell sorting (FACS) using a flow cytometer (BD Biosciences, San Jose, CA, USA). Written informed consents were obtained from all participants, and this study was approved by the research ethics committee of Wuhan University. The human MDS cell line (SKM-1), was donated by the Union Hospital of Huazhong University of Science and Technology, and leukaemia cell lines (K562, HL-60, THP-1) were kindly provided by the Institute of Hematology & Hospital of Blood Diseases Chinese Academy of Medical Sciences, China. SKM-1, K562, HL-60, and THP-1 cells were cultured in RPMI 1640 medium (Hyclone, Logan, UT, USA), while 293T cells were grown in Dulbecco's modified Eagle's medium (DMEM; Sigma-Aldrich, San Francisco, USA) supplemented with 10% foetal bovine serum (FBS; Gibco, MD, USA) and 2 mM L-glutamine and penicillin/streptomycin (Gibco, MD, USA). Human umbilical vein endothelial cells (HUVEC, from American Type Culture Collection [ATCC], Manassas, VA, USA) were maintained in Endothelial Cell Basal Medium (EBM) completed with 5 ng/mL recombinant human (rh) VEGF, 5 ng/mL rh epidermal growth factor (EGF), 5 ng/mL rh- basic fibroblast growth factor (b-FGF), 15 ng/mL rh- Insulin-like growth factor 1 (IGF-1), 10 mM l-glutamine, 0.75 units/mL heparin sulphate, 1 µg/mL hydrocortisone, 2% foetal bovine serum, and 50 µg/mL ascorbic acid. The cells were cultured in a humidified incubator at 37 °C with 95% air and 5% CO_2_.

### MiRNAs transfection

The miR-16 mimics (5ʹ-UAGCAGCACGUAAAUAUUGGCG-3ʹ), inhibitor (5ʹ-CGCCAAUAUUUACGUGCUGCUA-3ʹ), and negative control (5ʹ-UUCU CCGAACGUGUCACGUTT-3ʹ) with FAM fluorescence were purchased from GenePharma Co., Ltd. (Shanghai, China). Transfection of mimics or inhibitor was performed with Lipofectamine™ 2000 (Invitrogen, Thermo Fisher Scientific, Inc.) according to the manufacturer's instructions. Firstly, add 40-100pmol miR-16 mimics/inhibitor and 1µl lipofectamin reagent to the 50 µl DMEM serum free medium respectively, and mix them thoroughly in room temperature for 20 min, then 400µl cells suspension (about 4.0×10^5^ cells) were added to them and evenly distributed to 12 wells. Secondly, the cells were cultured in a humidified incubator at 37 °C with 95% air and 5% CO_2_. The transfection efficiency was about 90% as assessed by FACS. Cells were harvested 48 h-72 h after transfection for further analyses.

### MiRNAs RT-qPCR

Total RNA was extracted using TRIzol reagent (Invitrogen, Carlsbad, CA, USA) according to the manufacturer's instructions. cDNA was synthesised from total mRNA (2 μg) in 20μL reactions using a reverse transcriptase and miR-15a, miR-16, or U6 primers obtained from Invitrogen at 37 °C for 1 h. For measuring the expression of mature miR-15a and miR-16, a polyA qPCR analysis was performed. The primers specific for miR-15a and miR-16 were 5ʹ-UAGCAGCACAUAAUGGUUUGUG-3ʹ and 5ʹ- UAGCAGCACGUAAAUAUUGGCG-3ʹ, respectively. The reactions were performed in a 7500 Real-Time PCR System (Applied Biosystems, USA), using the U6 small nuclear RNA (specific primer: 5ʹ- CTCGCTTCGGCAGCACA-3ʹ) as an internal control. The qPCR reaction condition was performed by pre-denaturation (94˚C for 2 min) and denaturation with 45 cycles (94˚C for 2 min), annealing (62˚C for 34 sec), and extension (72˚C for 10 s) with 45 cycles. The relative expression was calculated using the 2^-ΔΔCt^ method 23. All qPCR assays were performed in triplicate.

### 3'-untranslated region (UTR) luciferase constructs generation and luciferase assays

Luciferase plasmids containing the wild-type or mutated miR-16 binding site in the 3'UTR of the VEGF mRNA was chemically synthesised by GenePharma Co., Ltd. (Shanghai, China). This sequence was cloned downstream of the stop codon in the psiCheck-2 vector (Promega, Madison, WI, USA) to generate the VEGF 3'UTR reporter. The primers used for the luciferase reporter plasmid construction are listed in Table 1. The 293T cells were grown in 24-well plates and co-transfected with luciferase reporters containing WT and mutant 3′-UTR of VEGF, and pre-miR-16 precursor or controls using Lipofectamine™ 2000, according to the manufacturer's instructions. Luciferase activity was measured using the Dual Luciferase Assay kit (Promega, USA). The relative luciferase activity was obtained by normalisation to the Renilla luciferase activity.

### Enzyme-linked immunosorbent assay (ELISA)

The levels of VEGF and other pro-angiogenic factors (b-FGF, connective tissue growth factor [CTGF], and matrix metalloproteinase-2 [MMP-2]) in culture supernatants were measured using specific ELISA kits (Neobioscience, Shenzhen, China) according to the manufacturer's instructions. Collected supernatants, blank control, and standard samples were transferred into a 96-well plate and incubated at 37 °C for 90 min, and then the supernatants were discarded, followed by washing with a washing buffer for 5 times. Biotinylated antibody was added into the wells of MDS and healthy controls, while Biotinylated antibody diluent was added into the blank control well and incubated at 37 °C for 60 min. The supernatants were then discarded, and the wells were washed 5 times. Enzyme conjugates were added and incubated at 37 °C for 30 min; the supernatants were then discarded, and the wells were washed 5 times. A chromogenic substrate was added and incubated at 37 °C for 15 min. Finally, a stop solution was added into the wells and the absorbance was measured using a microplate reader (Bio-Rad, USA) with optical density (OD) values at 450 nm.

### Western blotting analysis

Protein extraction and western blotting were performed as described previously 24. Briefly, 30-60 μg cell lysates were subjected to sodium dodecyl sulphate-polyacrylamide gel electrophoresis, transferred to nitrocellulose membranes, and blotted with a rabbit polyclonal anti-VEGF antibody (1:1,000, ab46154, Abcam, Burlingame, CA, USA) overnight at 4 ˚C. After washing three times with Tris-buffered saline with Tween-20 (TBST; Sigma-Aldrich, USA), the membranes were incubated with a horseradish peroxidase (HRP)-conjugated secondary antibody (1:1,000; sc-2012; Santa Cruz Biotechnology, Santa Cruz, CA, USA) at 37 ˚C for 2 h. The membranes were stripped using a western blot stripping buffer (Pierce, Rockford, IL, USA) and re-incubated with GAPDH (1:2,000; CST-#2118, Cell Signaling Technology, Danvers, MA, USA) to verify equal protein loading.

### Immunohistochemical staining (IHC)

Paraffin sections sliced from bone marrow specimens of MDS patients and healthy controls were deparaffinised twice for 10 min and then rehydrated with doubled distilled (dd)H_2_O. Subsequently, the slides were heated in 10 mM citrate buffer (pH 6.0) and treated with 3% H_2_O_2_ in phosphate-buffered saline (PBS) for 10 min, blocked with 5% normal goat serum, and incubated with a mouse monoclonal anti-VEGFA antibody (1:1,000; ab1316, Abcam, CA, USA) at 4 ºC overnight. Next, the slides were incubated with an HRP-Polymer-conjugated secondary antibody (Maixin, Shanghai, China) at 37 °C for 1 h. The slides were then stained with diaminobenzidine (DAB) for 3 min and counterstained with haematoxylin. Three fields were selected for examination with an inverted microscope (Olympus, Tokyo, Japan). The integrated optical density (IOD) of VEGF expressions were measured by Image-Pro Plus 6.0 software (Media Cybernetics, Siver Spring, MD, U.S.A.).

### Immunofluorescence (IF)

The number of cells in cell suspensions (K562, THP-1, SKM-1, and HL-60) was counted and adjusted to 1.5 × 10^4^/100 µL. Cells were centrifuged for 10 min at 450 g to allow their adhesion to the slides, and then washed three times with PBS (5 min/wash) and fixed with 4% paraformaldehyde for 15 min. The slides were then incubated with 0.25% bovine serum albumin for 5 min and 0.05%Triton X-100 in the slides for 5 min. After incubation with a rabbit polyclonal anti-VEGF antibody (1:1,000, ab46154, Abcam, CA, USA) overnight at 4˚C, the slides were incubated with a green fluorescence-labelled goat anti-rabbit secondary antibody (1:1000, sc-2012, Santa Cruz, CA, USA) for 60 min at 37 ˚C in the dark. Nuclei were stained with 4',6-diamidino-2-phenylindole (DAPI) in the dark for 10 min. The slides were then observed using a fluorescence microscope (Leica, Germany).

### HUVEC tube formation and cell migration assays

HUVECs tube formation on Matrigel (BD Biosciences, Bedford, MA, USA) was evaluated as previously described 25. At least, 30 min before the experiment, 48-well plates were coated with Matrigel (BD, Biosciences, USA) at 4 ºC. Next, trypsin-harvested HUVECs were seeded onto the Matrigel-coated plates (2 × 10^4^ cells per well) in serum-free EBM and incubated at 37 ˚C for 12 h. The supernatant of K562 cells overexpressing or silenced for miR-16, or medium from the control group was added to each well at a dilution of 1:1 (final volume/well, 500 μL). Images showing the formation of capillary-like structures were obtained after 12 h with an inverted microscope (Olympus, Tokyo, Japan) at 200× magnification.

Cell migration assays were performed using Transwells with 6.5 mm diameter and 8 μm pore size (Corning, Corning, NY, USA). HUVECs (1-2 × 10^4^ cells) were seeded in the upper chambers, while the lower chambers were filled with 500 µL of the supernatant of K562 cells overexpressing or silenced for miR-16 and incubated at 37 °C for about 36 h. The cells in the upper chamber were then removed using cotton swabs. Cells migrating into the bottom of the membrane were stained with 0.1% crystal violet for 20 min at 37 °C and washed with PBS. Four random fields from each membrane were photographed under an inverted microscope and counted for statistical analysis.

### Statistical analysis

All data are indicated as mean ± the standard deviation (SD). All results were analysed using the SPSS 24.0 software (IBM Corp., Armonk, NY, USA). One-way analysis of variance, or the Mann-Whitney U-test non-parametric alternative, were used for the analysis of differences between groups, as appropriate. Results were considered significantly different for *P* values < 0.05. All experiments were performed at least three times to ensure their reproducibility.

## Results

### VEGF levels are significantly higher and miR-16 is downregulated in higher-risk MDS

**Figure 1:**
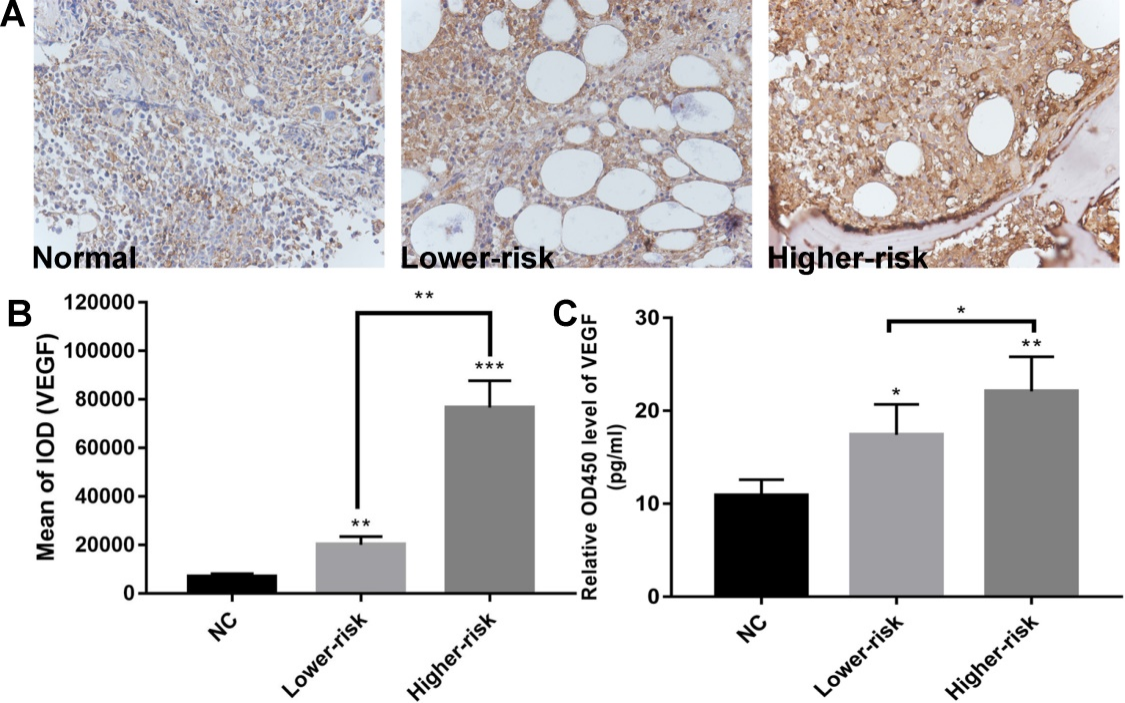
Aberrantly high expression of VEGF and down-regulated expression of miR-16 in high-risk MDS. (A) Representative immunohistochemical images of VEGF in MDS patients and controls. (B) Bar graph of the cumulative VEGF expression levels of different MDS risk patients (lower vs higher) vs healthy controls. Up-regulated expression of VEGF in the MDS patients compared with normal controls and stronger VEGF immunostaining in higher-risk MDS patients than in lower-risk MDS patients as revealed by immunohistochemistry. Staining was quantified by mean of integrated optical density (IOD). (C) ELISA reveals significantly higher VEGF secretion in MDS patients compared with normal controls and increased VEGF secretion in higher-risk MDS patients compared with lower-risk MDS. * *P*<0.05, **P<0.01, ***P<0.001.

**Figure 2:**
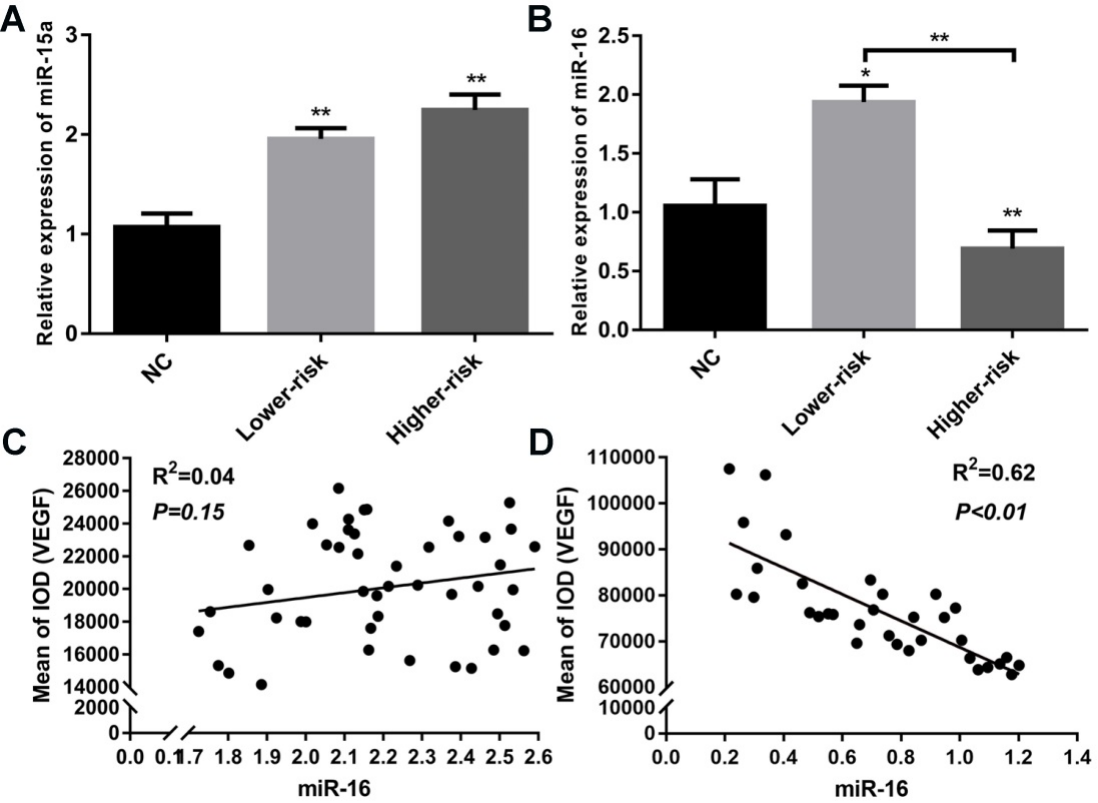
Aberrantly down-regulated expression of miR-16 and inverse correlation between miR-16 and VEGF expression in high-risk MDS. (A) CD34+cells from MDS patients and healthy controls were used to extract total RNA. RT-PCR of miR-15a revealed both lower-risk and higher-risk MDS patients had significantly higher miR-15a levels compared with normal controls. (B) RT-PCR of miR-16 revealed the expression level of miR-16 was remarkably lower in higher-risk MDS patients compared with normal controls and lower-risk patients. **(C)** Correlation analysis between miR-16 expression levels and VEGF expression levels in MDS lower-risk patients. **(D)** Correlation analysis between miR-16 expression levels and VEGF expression levels in MDS higher-risk patients. * *P*<0.05, **P<0.01.

MDS patients were classified into two large groups, a lower-risk group (very low and low IPSS-R risk) and a higher-risk group (intermediate, high, and very high IPSS-R risk) (n=47 and 33, respectively) according to the Revised International Prognostic Scoring System (IPSS-R)^26^ (Table 2). To identify the VEGF expression in MDS bone marrow samples, we performed IHC of VEGF on bone marrow biopsies. It showed increased expression of VEGF in MDS patients compared with healthy controls. And MDS patients with higher-risk exhibited a stronger positive immunostaining than MDS patients with lower-risk (Figure 1A-B). The secretion of VEGF in peripheral blood of MDS patients and controls was examined by ELISA. As shown in Figure 1C, VEGF secretion in patients with MDS (lower- and higher-risk) was higher than that in healthy subjects (*P* < 0.05 and *P* < 0.01, respectively) and VEGF secretion was significantly increased in the higher-risk group compared with the lower-risk group (*P* < 0.05). To investigate whether the upregulation of VEGF was associated with changes in miR-15a and miR-16 levels, we performed qPCR assays on freshly purified MDS CD34+ cells and normal CD34+ cells. The levels of miR-15a were significantly higher in patients with both lower-risk and higher-risk MDS than in healthy controls (*P* < 0.01). No significant difference in miR-15a levels was found between lower-risk and higher-risk MDS (Figure 2A). The levels of miR-16 were remarkably lower in higher-risk MDS patients and significantly higher in healthy individuals and lower-risk MDS patients (Figure 2B). Because higher-risk MDS patients have increased bone marrow angiogenesis, we hypothesised that the reduction in miR-16 levels might be involved in angiogenesis of MDS. Correlation analyses were performed to explore the relationships between miR-16 expression and VEGF expression on bone marrow biopsies in lower and higher-risk MDS patients (Figure 2C-D). Negative correlations between miR-16 levels and VEGF expression levels (R^2^=0.62, P<0.01) were determined in higher-risk MDS patients.

### Inverse correlation between VEGF and miR-16 in cells lines

**Figure 3:**
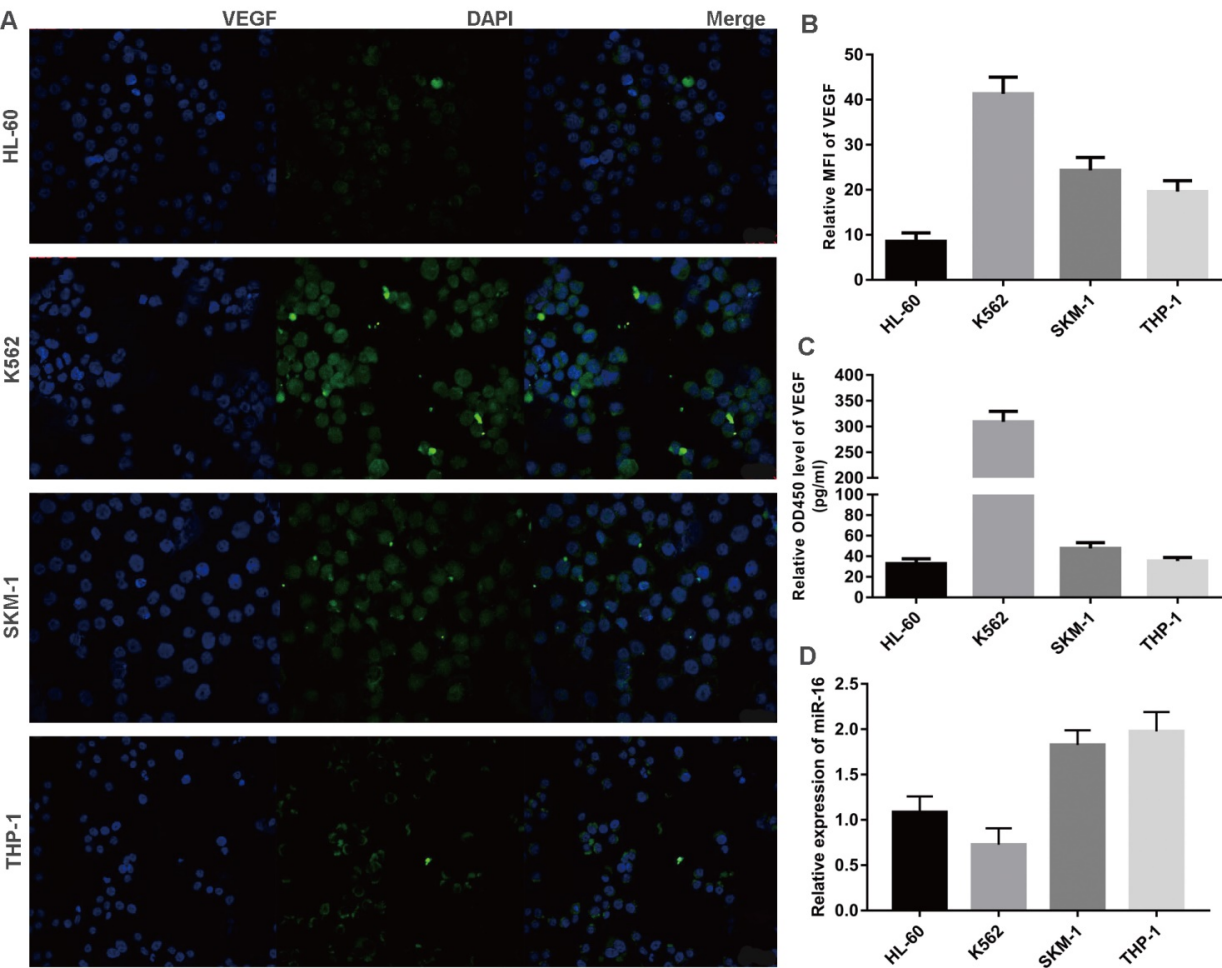
Inverse correlation between VEGF and miR-16 in cells lines (A) VEGF expression in four cell lines (K562, THP-1, HL-60 and SKM-1) were detected by immunofluorescence techniques. Representative images were taken to assess the expression level by confocal microscopy (×200 magnification). (B) MFI was measured by Volocity Demo6.1.1 software. (C) The secretion of VEGF in cell lines were tested by ELISA. (D) The miR-16 levels in cell lines were measured by qRT-PCR.

Three leukaemia cell lines (K562, THP-1, HL-60) and a MDS cell line (SKM-1) were analysed for VEGF and miR-16 expression. VEGF levels in exponentially growing cells were measured by immunofluorescence and the mean fluorescence intensity (MFI) was calculated, while the secretion of VEGF was determined by ELISA. As shown in Figure 3A-C, VEGF was expressed at high levels in K562 and SKM-1 cells. The levels of VEGF protein expression and secretion (mean ± SD) in the four cell lines were as follows (from high to low): K562, 40.36 ± 3.36 and 321.70 ± 20.29; SKM-1, 26.73 ± 1.66 and 48.93 ± 6.63; THP-1, 20.86 ± 0.50 and 35.74 ± 4.88; and HL-60, 8.97 ± 1.63 and 33.30 ± 6.96). qPCR showed that the levels of miR-16 (mean ± SD) were as follows (from low to high): K562, 0.72 ± 0.27; HL-60, 1.12 ± 0.16; SKM-1, 1.83 ± 0.13; and THP-1, 1.92 ± 0.16 (Figure 3D). These data indicate that the levels of VEGF are inversely correlated with those of miR-16 in SKM-1, THP-1, and K562 cells, but not in HL-60 cells.

### VEGF expression is inhibited by miR-16

**Figure 4:**
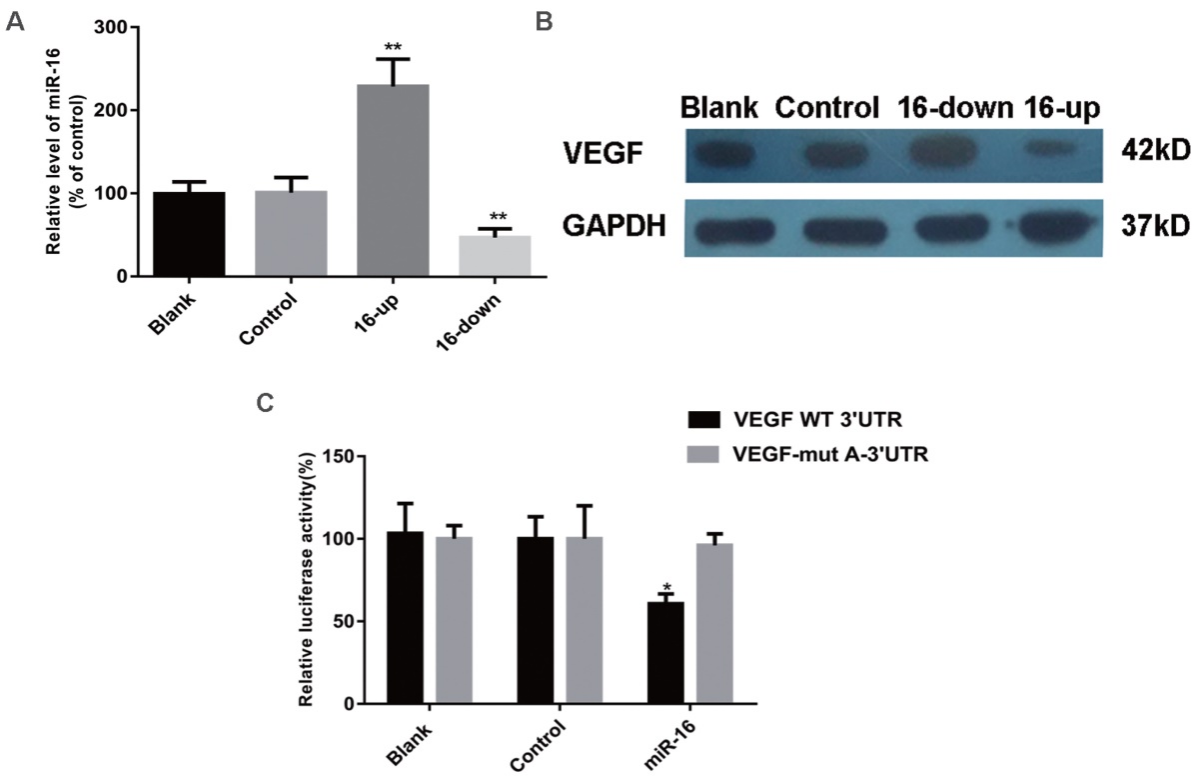
VEGF is a direct target of miR‑16 in cells. (A) The miR-16 levels of SKM-1 cells following miR-16 mimics /inhibitor (16-up/down) transfection for 48 h were analysed by qPCR. (B) The expression of VEGF protein of SKM-1 cells following miR-16 mimics /inhibitor transfection for 48 h was analysed by Western blotting analysis with β-actin as internal control. (C) Luciferase reporter assays were performed by co-transfection of pre-miR-16 oligonucleotide with a luciferase reporter gene containing wild-type 3'UTR of VEGF or mutated 3'UTR of VEGF. Expression of pre-miR-16 reduced luciferase activity of the wild-type reporter. * *P*<0.05, **P<0.01.

To determine whether the secretion of VEGF and other pro-angiogenic factors (b-FGF, CTGF, and MMP-2) were altered by miR-16, SKM-1 cells were transfected with a specific miR-16 inhibitor, mimics, or control (empty) particles. MiR-16 levels at 48 h after transfection were detected by qPCR (Figure 4A). ELISA was performed 48 h after transfection to analyse the secretion of VEGF, b-FGF, CTGF, and MMP-2. As shown in Table 3, VEGF levels were significantly decreased in the supernatant of miR-16-overexpressing cells (*P* < 0.001) and increased in that of miR-16-knock down cells (*P* < 0.01) compared with those in non-transfected cells or cells transfected with control miRNA. However, the secretion of b-FGF, CTGF, and MMP-2 did not significantly change among the different groups.

To examine whether VEGF is indeed a target of miR-16, its expression was evaluated by western blotting in SKM-1 cells as described above. VEGF levels were significantly reduced in cells overexpressing miR-16, compared with those in non-transfected cells or cells transfected with control miRNA. In contrast, knockdown of miR-16 was associated with increased expression of VEGF (Figure 4B). These results strongly suggested that VEGF may be a target of miR-16 in MDS cells.

### The 3ʹ-UTR of VEGF is a target of miR-16

A luciferase reporter assay was used to verify the direct interaction between miR-16 and the 3ʹ-UTR of VEGF. Wild-type (WT) or mutant (MUT) VEGF 3ʹ-UTR, in which the putative miR-16 binding site has been altered, was cloned into reporter plasmids. 293T cells were co-transfected with pre-miR-16 precursor or control and either the WT or MUT VEGF 3ʹ-UTR constructs. The overexpression of miR-16 markedly decreased the luciferase activity of the wild-type reporter but not that of the mutant reporter (*P* < 0.05), suggesting that miR-16 targets the 3ʹ-UTR of VEGF and that the point mutations in MUT VEGF 3ʹ-UTR abolish its effect (Figure 4C).

### miR-16 negatively regulates HUVEC tube formation and migration *in vitro*

**Figure 5:**
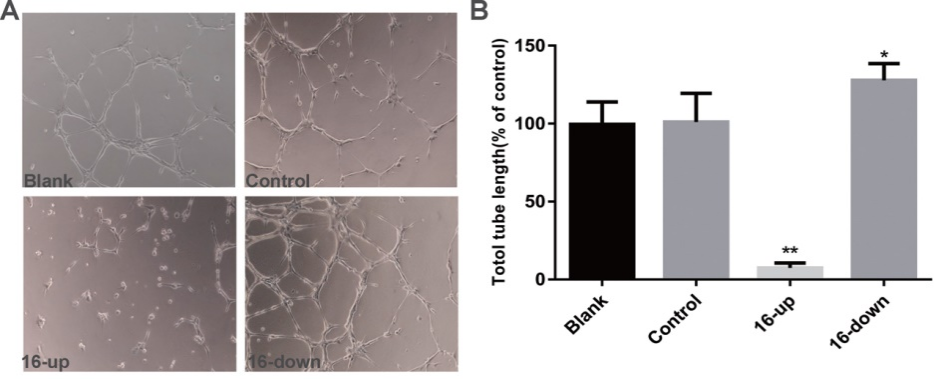
miR-16 negatively regulates HUVEC tube formation *in vitro* (A) Tube-formation assays with HUVECs were performed with the different groups of conditioned medium from K562 cells transfected with miR-16 inhibitor (16-down), miR-16 mimics (16-up) or empty vector. Representative images were taken to assess the tube formation (×200 magnification). (B) Quantification of the vascular tube structure was conducted by measuring the total tubule length in 5 high-power fields. *P<0.05. ** *P*<0.01.

**Figure 6:**
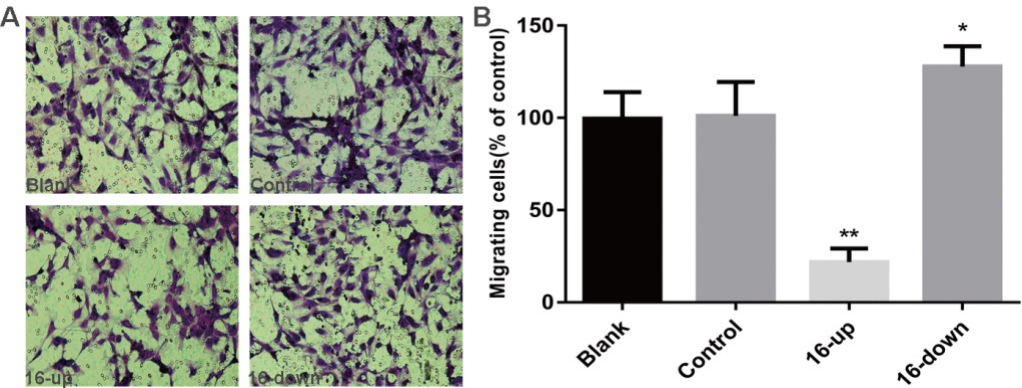
miR-16 negatively regulates HUVEC migration *in vitro* (A) Transwell migration assays with HUVECs were performed with the different groups of conditioned medium from K562 cells transfected with miR-16 inhibitor (16-down), miR-16 mimics (16-up) or empty vector. Representative images were taken to assess the cell migration in the open space (×200 magnification). (B) Quantification of cell migration capability was evaluated by measuring the distances migrated in 5 high-power fields. Data represent the mean±SD from three independent experiments. *P<0.05. ** *P*<0.01.

To investigate the impact of miR-16 on tumour angiogenesis, we performed tube-formation assays and cell migration assays. HUVEC tube formation assays were performed using three groups of conditioned medium of transfected SKM-1 cells: miR-16 inhibitor, mimics, and control. Our data revealed that the tube-forming capability of HUVECs was suppressed by medium from cells transfected with miR-16 mimics (*P* < 0.01), and opposite results were obtained upon transfection with the miR-16 inhibitor (*P* < 0.05, Figure 5A-B). Next, Cell migration assays on HUVECs were performed using conditioned medium of the above three groups. Compared with control cells, medium from cells overexpressing miR-16 dramatically suppressed the migration of HUVECs (*P* < 0.01), whereas the medium of cells with miR-16 knock down augmented the migration of HUVECs (*P* < 0.05, Figure 6A-B). These results demonstrated that miR-16 significantly affects the tube formation ability and the migration ability of HUVEC cells *in vitro*.

## Discussion

Using ELISA and IHC, we confirmed that VEGF secretion in the peripheral blood and VEGF levels in the bone marrow of MDS patients were significantly increased in higher-risk group compared with those in lower-risk group according to the IPSS-R classification. Although previous studies also demonstrated that increased expression of VEGF is associated with high-risk MDS and predicted decreased survival 11-14, the role of VEGF in MDS progression is yet to be clarified. Meanwhile, no study has investigated the mechanism of the significant differential expression of VEGF between high and low-risk MDS. In the present study, we investigated the possible role of miR-15a and miR-16 in the regulation of VEGF and their effect on angiogenesis in MDS.

In humans, miR-15a and miR-16 are clustered within 0.5 kb at chromosome 13q14, which is frequently deleted or downregulated in chronic lymphocytic leukaemia (CLL) 27, 28. Previous reports have demonstrated that the miR-15a/16 cluster functions as a tumour suppressor in CLL 28, 29, prostate cancer 30, and non-small cell lung cancer 31. Pons* et al.* reported that miR-15a is overexpressed in the bone marrow of patients with IPSS high-risk MDS and that miR-16 is overexpressed in the peripheral blood of IPSS lower-risk MDS 32. However, few studies have investigated the function of miR-15a/16 in MDS. In this study, we investigated the expression levels of miR-15a and miR-16 in bone marrow CD34+ cells of MDS patients and analysed the correlation between their expression and disease risk. Our results showed that miR-15a is similarly upregulated in both lower-risk and higher-risk MDS patients compared with healthy controls and that there is no difference in miR-15a level between lower-risk and higher-risk MDS. However, decreased miR-16 levels were observed in higher-risk MDS patients, and miR-16 levels were significantly higher in healthy individuals and lower-risk patients. Detection of VEGF and miR-16 levels in K562, THP-1 and SKM-1 cells also confirmed the inverse correlation between VEGF and miR-16 in MDS and leukaemia. Although several miRNAs have been implicated in the proliferation and differentiation of haematopoietic cells in MDS 33-36, there are few published data regarding the role of miRNAs in the angiogenesis of MDS. Because higher-risk MDS patients have increased bone marrow angiogenesis, we speculate that downregulation of miR-16 contributes to diseases progression by inducing angiogenesis in high-risk MDS.

Then, we demonstrated that ectopic expression of miR-16 efficiently inhibits the secretion of VEGF but does not affect that of other pro-angiogenic factors. Luciferase reporter assays confirmed that miR-16 binds to the VEGF 3′-UTR. Furthermore, the ectopic expression of miR-16 inhibits the overexpression of VEGF in SKM-1 cells and negatively regulates the migration and tube formation ability of HUVEC cells *in vitro*. Similar to all other miRNAs, miR-15a/16 has a broad range of cellular targets 37-39. B-cell lymphoma 2 (Bcl-2) has been reported as one of the targets of miR-15a/16 in CLL and prostate cancer, and inhibition of cell proliferation by miR-15a/16 through Bcl-2 has been reported in both lymphoid and non-lymphoid tissues 29, 40. This study highlighted a distinct role of miR-16 in angiogenesis in high-risk MDS via VEGF.

Although recent studies have revealed that VEGF expression is closely correlated with the progression of MDS, advancements in measures of dealing with the poor prognosis of high-risk MDS have been limited, with no significant change in recent years 41. Notably, the clinical use of anti-VEGF agents is limited because of their adverse effect on normal vasculature 42. Re-expression of lost miRNAs has attracted much attention as a promising strategy in targeted therapy of tumours 43. We believe that miR-16 may serve as a potential diagnostic marker and therapeutic target in high-risk MDS patients. However, additional studies are necessary to investigate the detailed regulatory mechanisms of miR-16 in high-risk MDS.

In summary, we demonstrated that miR-16 expression is downregulated in patients with high-risk MDS and that miR-16 negatively modulate angiogenesis via VEGF suppression. These evidences suggest that miR-16 may act as a tumour suppressor in MDS development, by directly targeting VEGF.

## Figures and Tables

**Table 1:** Primers used for luciferase reporter plasmid construction

Primer name	Sequence oligonucleotide (5'-3')
VEGF-UTR-Forward	TAGGCGATCGCTCGAGGCCGGGCAGGAGGA A
VEGF-UTR-Reverse	AATTCCCGGGCTCGAGTGAGATCAGAATTA
VEGF-Mut(16)-Forward	TTCTACGACGAAAATCACCGAGCCCGGAAG
VEGF-Mut(16)-Reverse	ATTTTCGTCGTAGAAAAATAAAATGGCGAATCC

VEGF, Vascular endothelial growth factor; UTR, untranslated region; Mut, mutant.

**Table 2:** Clinical characteristics of MDS patients

Features	
No. of patients	80
Sex, M/F	41/39
Age(years), median (range)	53 (24-82)
WBC (10^9^/L), median (range)	3.5 (0.5-15.9)
Neutrophils(10^9^/L), median (range)	0.9 (0-8.1)
Hemoglobin (g/L), median (range)	68 (40-153)
RBC (10^12^/L), median (range)	2.7 (3.1-5.6)
Platelets (10^9^/L), median (range)	64 (5-571)
MCV (fL), median (range)	101 (70-125)
WHO subgroups	
MDS-SLD	14(17.5%)
MDS-MLD	32 (40.0%)
MDS-U	11(13.8%)
MDS-EB-I	12 (15.0%)
MDS-EB-II	11 (13.8%)
IPSS-R risk groups	
Very Low	12(15.0%)
Low	35 (43.7%)
Intermediate	13 (16.3%)
High	12 (15.0%)
Very High	8 (10.0%)

WBC: white blood cells; RBC: red blood cell; MCV: mean corpuscular volume; MDS-SLD: MDS with single lineage dysplasia; MDS-MLD: MDS with multilineage dysplasia; MDS-U: MDS-unclassifiable; MDS-EB-1: MDS with excess blasts-1; MDS-EB-2: MDS with excess blasts- 2; IPSS-R: Revised-International Prognostic Scoring System.

**Table 3:** Secretion of angiogenic factors from control and transfected SKM-1 cells

CM of SKM-1 cells	VEGF (pg/10^6^ cells/ml) (mean ± SD)	bFGF (pg/10^6^ cells/ml) (mean ± SD)	CTGF (pg/10^6^ cells/ml) (mean ± SD)	MMP-2(pg/10^6^ cells/ml) (mean ± SD)
Non-transfected cells	82.7 ±3.1	62.1±2.9	1.61±0.28	10.2±0.9
miRNA precursor control	83.4 ±3.2	68.9±3.4	1.65±0.09	11.1±1.3
mimics-miR-16	30.9±4.9***	63.6±3.4	1.51±0.29	10.8±1.9
inhibitor-miR-16	102.7±6.7**	62.9±3.9	1.75±0.22	10.4±0.8

(***P<0.001, **P<0.01) CM: culture media; VEGF: Vascular endothelial growth factor; b-FGF: basic fibroblast growth factor; CTGF: connective tissue growth factor; MMP-2: matrix metalloproteinase-2.
